# Helicobacter pylori infection and small intestinal bacterial overgrowth: a systematic review and meta-analysis

**DOI:** 10.1186/s12866-023-03063-w

**Published:** 2023-12-06

**Authors:** Liang Liao, Bin-Bin Su, Shi-Ping Xu

**Affiliations:** Department of Gastroenterology, The Second Medical Center of PLA General Hospital, No.28 Fuxing Road, Beijing, 100853 China

**Keywords:** Small intestinal bacterial overgrowth, *Helicobacter pylori*, Proton pump inhibitor, Gastrointestinal symptoms, Meta-analysis

## Abstract

**Background:**

There is a link between *Helicobacter pylori* (HP) infection and small intestinal bacterial overgrowth (SIBO) with nonspecific digestive symptoms. Nonetheless, whether HP infection is associated with SIBO in adults remains unclear. Based on a meta-analysis, we evaluated this relationship.

**Results:**

Observational studies relevant to our research were identified by searching PubMed, Embase, the Cochrane Library, and the Web of Science. We evaluated between-study heterogeneity using the Cochrane Q test and estimated the I^2^ statistic. Random-effects models were used when significant heterogeneity was observed; otherwise, fixed-effects models were used. Ten datasets from eight studies, including 874 patients, were involved in the meta-analysis. It was shown that HP infection was related to a higher odds of SIBO (odds ratio [OR]: 1.82, 95% confidence interval: 1.29 to 2.58, *p <* 0.001) with mild heterogeneity (*p* for Cochrane Q test = 0.11, *I*^*2*^ = 7%). Subgroup analyses showed that HP infection was related to SIBO in young patients (mean age < 48 years, OR: 2.68, 95% CI: 1.67 to 4.28, *p <* 0.001; *I*^*2*^ = 15%) but not in older patients (mean age ≥ 48 years, OR: 1.15, 95% CI: 0.69 to 1.92, *p <* 0.60; *I*^*2*^ = 1%; p for subgroup difference = 0.02). Subgroup analyses further indicated that the association was not significantly affected by the country of study, comorbidities, exposure to proton pump inhibitors, or methods of evaluating HP infection and SIBO.

**Conclusions:**

HP infection may be related to SIBO in adults, which supports the detection of SIBO in patients with digestive symptoms and HP infection.

## Background

Physically, the small intestine has relatively low-level colonized bacteria compared to the colon [1]. Recent evidence from preclinical and clinical studies suggests that excessive bacterial growth in the small intestine, which is called small intestinal bacterial overgrowth (SIBO) [2], may be an underlying pathophysiological change of various unspecific gastrointestinal (GI) symptoms and the mechanisms of pathogenesis of various digestive and other systematic diseases [3]. Indeed, accumulating evidence suggests that SIBO is not only observed in patients with irritable bowel syndrome (IBS) [4], functional dyspepsia [5], inflammatory bowel disease [4], chronic pancreatitis [6], and liver cirrhosis [7], but also in those with non-alcoholic fatty liver disease [8], diabetes [9], systemic sclerosis [10], and Parkinson’s disease [11] etc. These observations suggest that SIBO may be an essential pathophysiological process involved in the pathogenesis and progression of these disorders [12, 13]. However, the potential mechanisms of SIBO in these clinical conditions are still to be determined.

*Helicobacter pylori* (HP) infection is also a risk factor for various gastric diseases, such as gastric and duodenal ulcer, atrophic gastritis, and gastric cancer [14, 15]. Increasing studies suggest that besides gastric diseases, HP infection may also be involved in the pathogenesis of some intestinal disorders [16]. For example, HP infection has been linked to the risk of colorectal adenomas [17] and colorectal cancer [18], as well as functional disorders such as IBS [19], suggesting a close relationship between HP infection and disturbed intestinal homeostasis. According to previous studies, impaired gastric motility and/or acidity will likely boost bacterial growth in the small intestine and increase colonization [20, 21]. As a gram-negative bacterium, hydrolysis of urea by HP leads to ammonia and carbonic acid, which buffers gastric acid and maintains the proliferation of intestinal organisms [20, 21]. Further, long-term HP infection may also lead to atrophies in the gastric mucosa, facilitating the growth of intestinal bacteria [20, 21]. Therefore, it could be hypothesized that there may be a link between HP infection and SIBO [16]. Despite this, previous studies have not been able to establish a causal relationship between HP infection and SIBO in the adult population [22]. Therefore, we conducted a meta-analysis to determine whether SIBO in adults is associated with HP infection.

## Results

### Database search and study retrieval

**Fig. 1:**
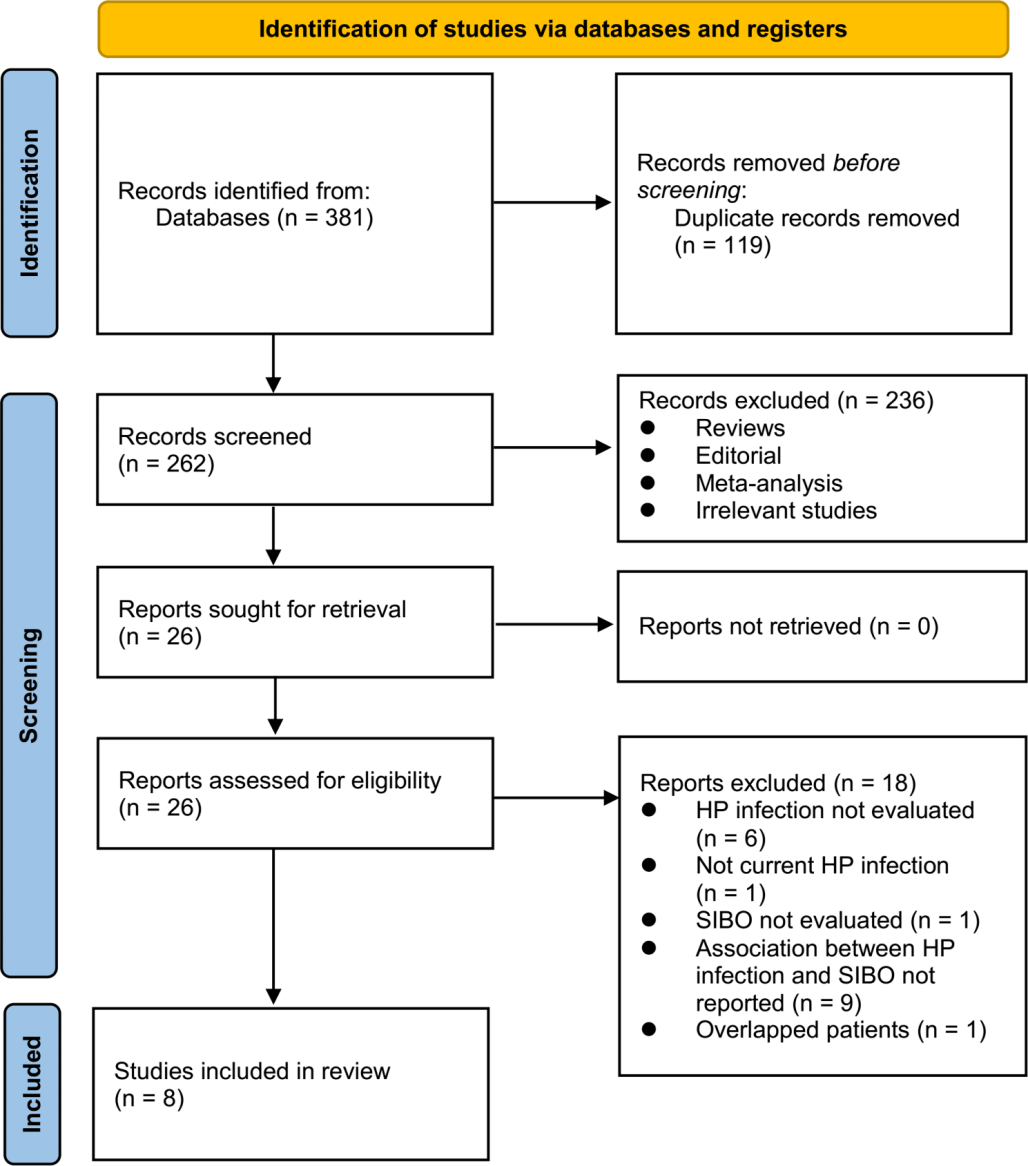
Flowchart of database search and study inclusion

Figure 1 shows the process of literature search and study retrieval. Initially, 381 records were obtained from the database, and 119 duplicate entries were removed. Afterward, 236 studies were removed based on the title and abstract screening as they did not fit the meta-analysis’s objectives. Following full-text reviews of 26 studies, 18 were excluded for the reasons listed in Fig. 1. Accordingly, eight studies were obtained for subsequent meta-analysis [23–30].

### Study characteristics

One of the studies reported two datasets in patients with rosacea or skin naevi separately [24]. Another study reported two datasets in patients with and without diabetes separately [28]. Accordingly, these datasets were independently incorporated into the meta-analysis. Ten datasets from eight studies [23–30], which included 874 patients, were used for the meta-analysis. The characteristics of the included studies are summarized in Table 1. These were all cross-sectional studies published between 2013 and 2022 and performed in Italy, Austria, India, Ukraine, Korea, and China. Six datasets included patients with GI symptoms with no other comorbidities [25–30], while the other four datasets included patients with Parkinson’s disease, rosacea, skin naevi, or diabetes [23, 24, 28]. Patients with recent use of antibiotics were excluded from all the included studies. Six studies also excluded patients with recent use of PPIs [23–26, 29, 30], while the other two studies did not specify the recent use of PPIs [27, 28]. In these studies, the average age of the participants was 42 to 68 years old, with a proportion of 30–69% males. For the detection of HP infection, UBT [23–27, 30], SAgT [28], and RUT [29] were used, which showed an overall prevalence of HP infection of 42.6% (373/874). For the evaluation of SIBO, LBT [23, 25], GBT [24, 26, 28–30], and QDC [27] were used, which suggested an overall prevalence of SIBO of 30.3% (265/874). All included studies scored between seven and eight stars, indicating moderate to good quality (Table 2).

**Table 1:** Characteristics of the included studies

Study	Design	Country	Diagnosis	Sample size	Mean age (years)	Men (%)	HP evaluation	HP positive	SIBO evaluation	SIBO positive
Fasano 2013	CS	Italy	PD patients without GI disease, no exposure to PPI or antibiotics	33	68.1	58.1	UBT	11	LBT	18
Gravina 2015a	CS	Italy	Patients with rosacea, no exposure to PPI or antibiotics	90	51.5	43.3	UBT	44	GBT	9
Gravina 2015b	CS	Italy	Patients with skin naevi, no exposure to PPI or antibiotics	90	48	47.8	UBT	24	GBT	7
Del Zompo 2016	CS	Italy	Patients with GI symptoms, no exposure to PPI or antibiotics	136	42.5	30.1	UBT	36	LBT	17
Enko 2017	CS	Austria	Patients with GI symptoms, no exposure to PPI or antibiotics	109	44	33	UBT	36	GBT	35
Mujeeb 2019	CS	India	Patients with GI symptoms undergoing UGIE, no exposure to antibiotics	80	45.4	75.2	UBT	28	QDC	19
Radionova 2020a	CS	Ukraine	Diabetic patients with chronic active gastritis, no exposure to antibiotics	92	61.6	68.5	SAgT	71	GBT	69
Radionova 2020b	CS	Ukraine	Non-diabetic patients with chronic active gastritis, no exposure to antibiotics	80	54	57.5	SAgT	48	GBT	33
Kim 2022	CS	Korea	Patients with GI symptoms undergoing UGIE, no exposure to PPI or antibiotics	62	49.8	30.6	RUT	22	GBT	11
Zhu 2022	CS	China	Patients with GI symptoms, no exposure to PPI or antibiotics	102	42.1	48	UBT	53	GBT	47

HP: helicobacter pylori; SIBO: small intestinal bacterial overgrowth; GI: gastrointestinal; PD: Parkinson’s disease; PPI: pronto pump inhibitor; UGIE: upper gastrointestinal endoscopy; UBT: urea breath test; SAgT: stool antigen test; RUT: rapid urease test; LBT: lactulose breath test; QDC: quantitative duodenal aspirate culture; GBT: glucose breath test;

**Table 2:** Study quality evaluation via the Newcastle-Ottawa Scale

Study	Adequate definition of cases	Representativeness of cases	Selection of controls	Definition of controls	Control for age and sex	Control for other confounders	Exposure ascertainment	Same methods for events ascertainment	Non-response rates	Total
Fasano 2013	1	1	1	1	0	0	1	1	1	7
Gravina 2015a	1	1	1	1	0	0	1	1	1	7
Gravina 2015b	1	1	1	1	0	0	1	1	1	7
Del Zompo 2016	1	1	1	1	1	0	1	1	1	8
Enko 2017	1	1	1	1	0	0	1	1	1	7
Mujeeb 2019	1	1	1	1	0	0	1	1	1	7
Radionova 2020a	1	1	1	1	0	0	1	1	1	7
Radionova 2020b	1	1	1	1	0	0	1	1	1	7
Kim 2022	1	1	1	1	0	0	1	1	1	7
Zhu 2022	1	1	1	1	0	0	1	1	1	7

### Meta-analysis results

**Fig. 2:**
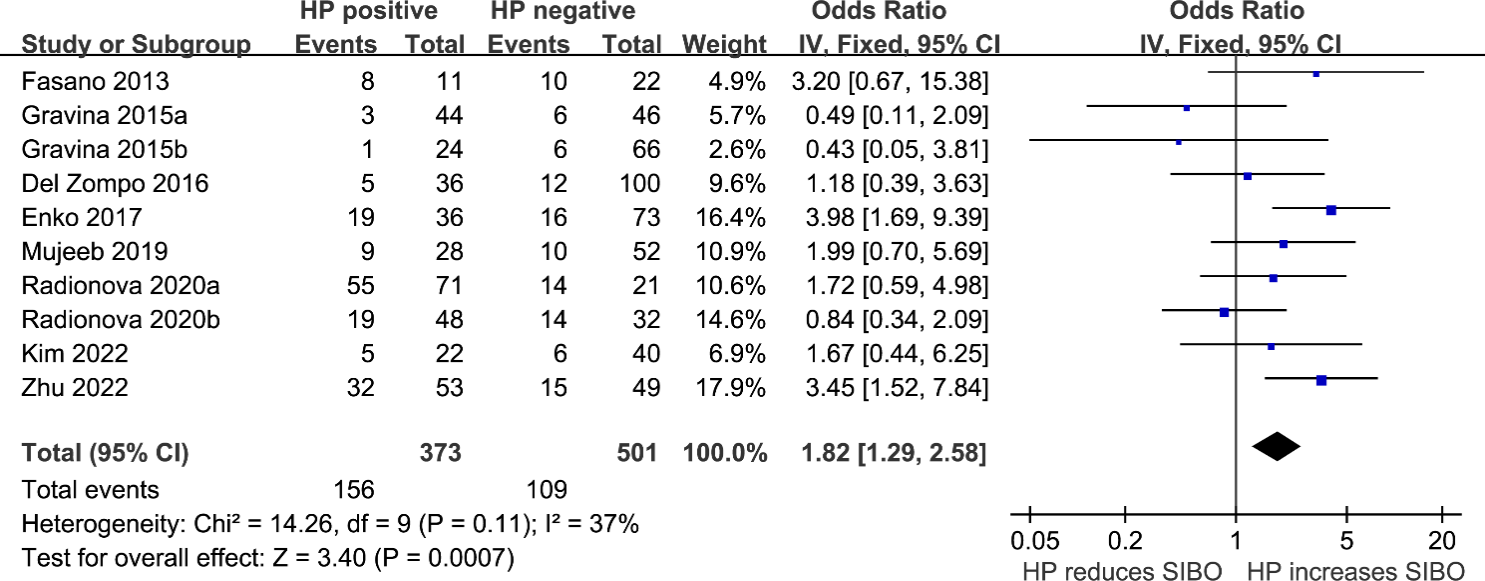
Forest plots for the meta-analyses regarding the association between HP infection and SIBO in adult patients

**Fig. 3:**
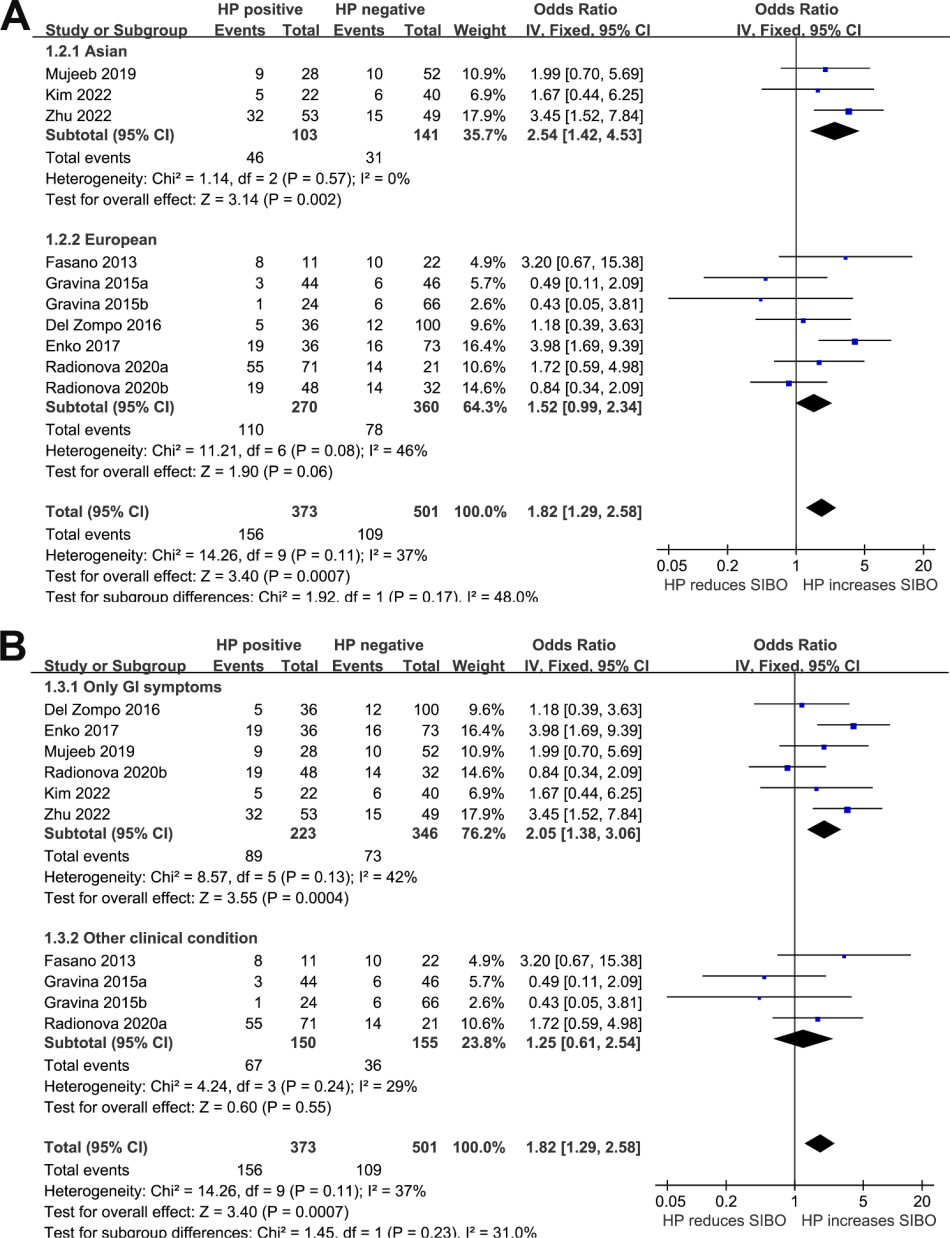
Forest plots for the subgroup analyses regarding the association between HP infection and SIBO in adult patients; **A** subgroup analysis according to study country; and **B** subgroup analysis according to the comorbidities of the patients

**Fig. 4:**
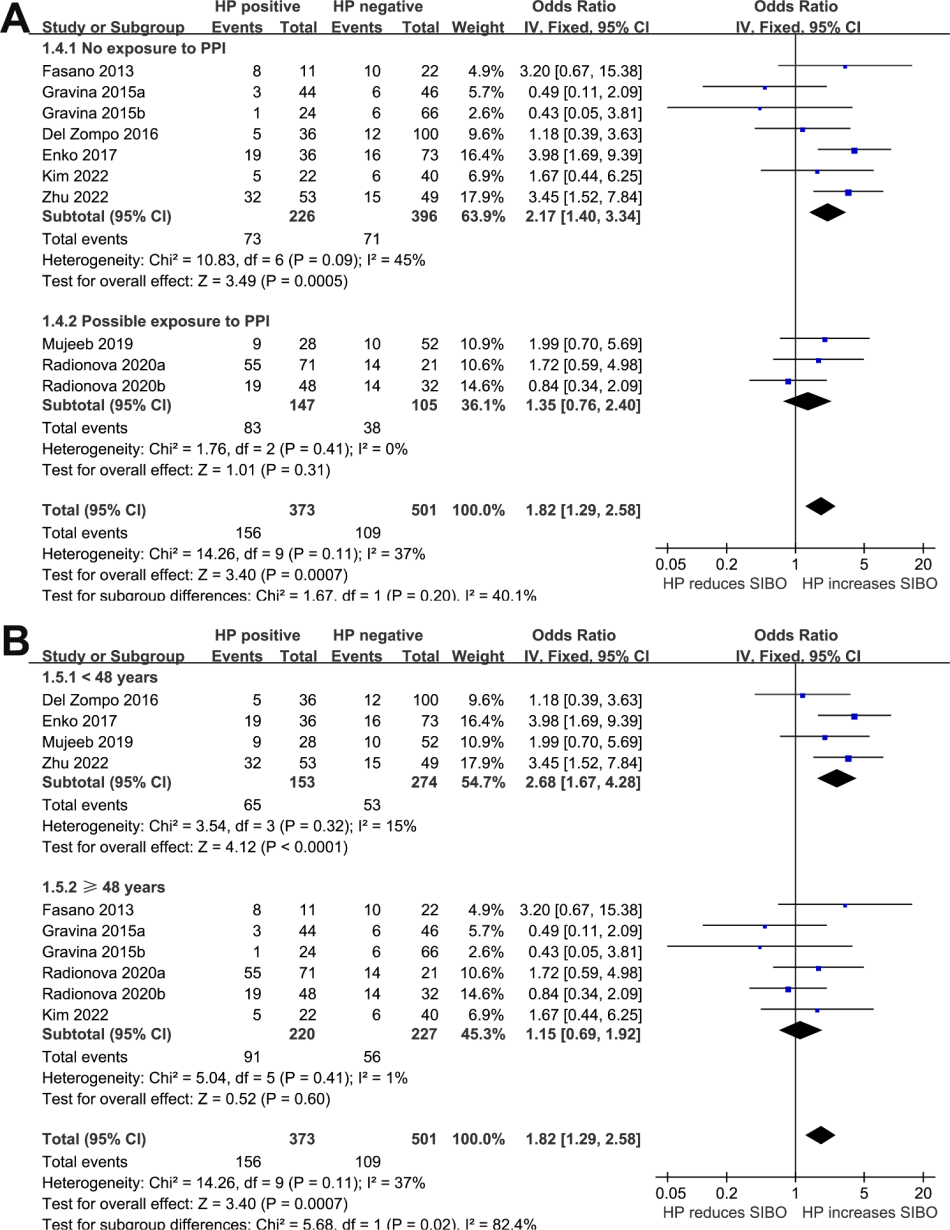
Forest plots for the subgroup analyses regarding the association between HP infection and SIBO in adult patients; **A** subgroup analysis according to the possible PPI exposure; and **B** subgroup analysis according to the mean ages of the patients

**Fig. 5:**
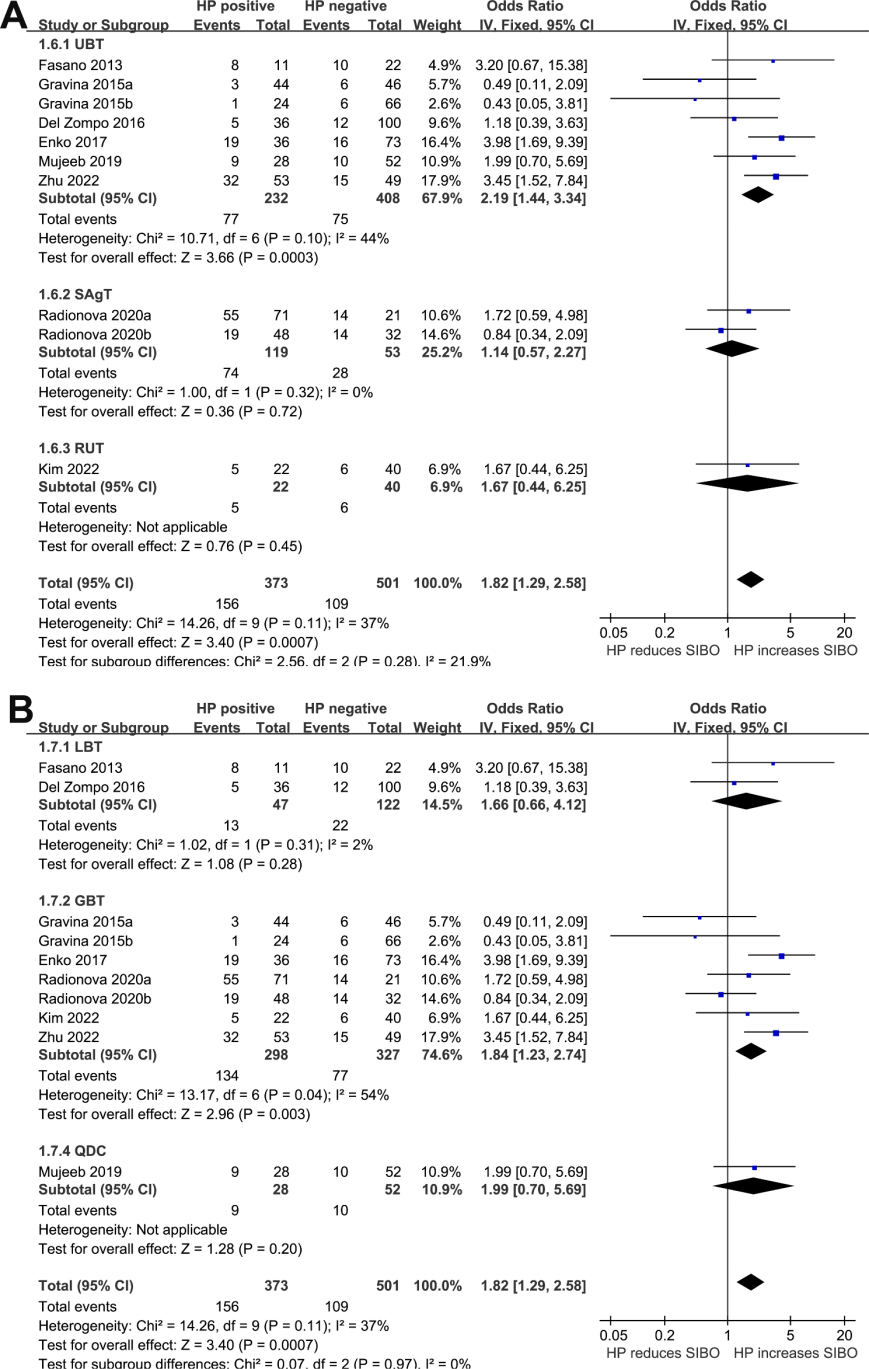
Forest plots for the subgroup analyses regarding the association between HP infection and SIBO in adult patients; **A** subgroup analysis according to the methods for detecting HP infection; and **B** subgroup analysis according to the methods for evaluating SIBO.

We detected a non-significant heterogeneity (p for Cochrane Q test = 0.11, *I*^*2*^ = 37%) among the included studies, and a fixed-effects model was used for the meta-analysis. Pooled results showed that HP infection was related to higher odds of SIBO (OR: 1.82, 95% CI: 1.29 to 2.58, *p <* 0.001; Fig. 2). Sensitivity analyses omitting one dataset at a time also retrieved consistent results (OR: 1.57 to 2.08, p all < 0.05). Subgroup analyses suggested that the association between HP infection and SIBO was not significantly influenced by study country (Fig. 3A), comorbidities (Fig. 3B), or possible exposure to PPIs (Fig. 4A), with between-subgroup p values all > 0.05. Interestingly, subgroup analysis showed that the association between HP infection and SIBO was significant in younger patients (mean age < 48 years, OR: 2.68, 95% CI: 1.67 to 4.28, *p <* 0.001; *I*^*2*^ = 15%) but not in older patients (mean age ≥ 48 years, OR: 1.15, 95% CI: 0.69 to 1.92, *p <* 0.60; *I*^*2*^ = 1%; p for subgroup difference = 0.02; Fig. 4B). Furthermore, subgroup analyses suggested that different methods of detecting HP infection and SIBO did not significantly affect the association (p for subgroup differences both > 0.05, as shown in Fig. 5**(A and B**).

### Publication bias

**Fig. 6:**
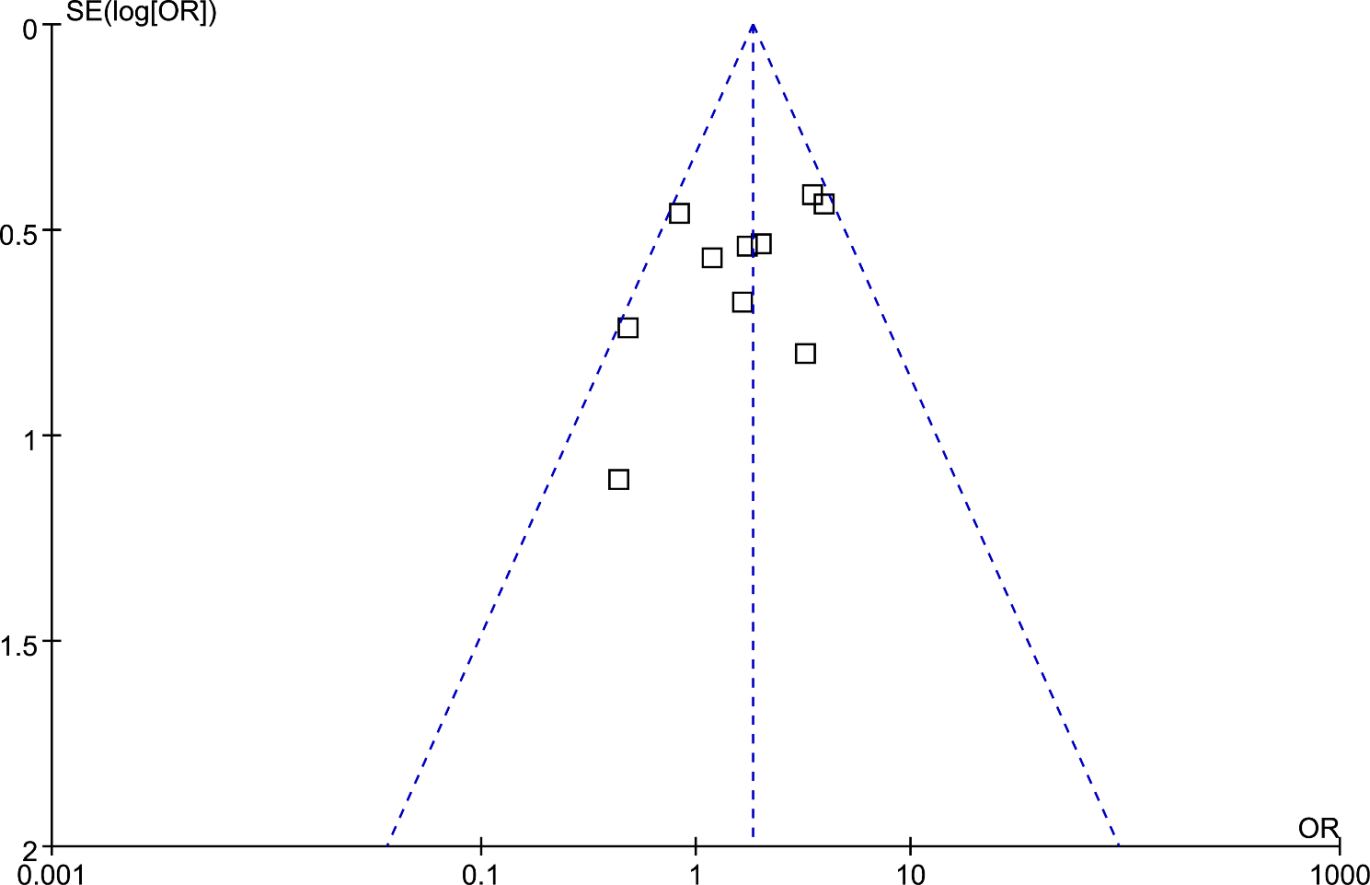
Funnel plots for the publication bias underlying the meta-analysis regarding the association between HP infection and SIBO in adult patients

The funnel plots for the meta-analysis of HP infection and SIBO in adult patients are presented in Fig. 6. Based on visual examination, the plots are symmetrical, suggesting low publication bias. Additionally, Egger’s regression tests indicated a low likelihood of publication bias (*p* = 0.81).

## Discussion

In this systematic review and meta-analysis, we pooled the results of eight eligible observational studies and found that compared to adults without HP infection, those with HP infection were associated with a higher prevalence of SIBO. Consistent results were achieved in sensitivity analyses, omitting each study at a time as well as subgroup analysis according to study country, comorbidities, possible exposure to PPI, and methods for the detection of HP infection and SIBO. Interestingly, we found that the association between HP infection and SIBO was stronger in younger than older patients. The findings suggest that HP infection is associated with SIBO in adults, especially in younger individuals.

As far as we know, this is the first meta-analysis investigating the potential association between HP infection and SIBO prevalence among adults. The methodological advantages of the study included the following. A comprehensive literature search was conducted in four commonly used databases, which could provide current evidence regarding this link between HP infection and SIBO. Second, all of the included studies enrolled patients without exposure to recent antibiotics, which excluded the potential confounding effects of antibiotics on SIBO prevalence. This is important because it has been suggested that antibiotics such as rifaximin may effectively eradicate SIBO, which may affect the meta-analysis results [31]. As a final step, multiple sensitivity analyses and subgroup analyses were conducted, and these consistent results further confirmed the findings’ stability. Results from these studies support the idea that HP infection is associated with SIBO in adults. The findings support the hypothesis that HP infection impairs gastric motility and buffers gastric acid to increase the colonization and proliferation of intestinal bacteria [16].

Based on our subgroup analysis, HP infection and SIBO are consistently associated in patients without and possibly exposed to PPIs. This is important because using PPIs is associated with an increased risk of SIBO [32], probably due to the increased gastric PH following the medications. Therefore, using PPIs may confound the association between HP infection and SIBO. Our findings showed a consistent result in patients without exposure to PPIs, suggesting that the association between HP infection and SIBO was independent of using PPIs in these patients. Interestingly, the subgroup analysis results according to the patients’ age showed that the association between HP infection and SIBO was significant in young but not in older patients. The mechanisms underlying these findings are not unknown. From our point of view, older people are likely to have multiple comorbidities, which may also affect the SIBO in these patients besides the HP infection status. On the other hand, these results may highlight the importance of detecting SIBO in younger patients with HP infection. For these patients, particularly for those with unspecific digestive symptoms, besides HP infection, SIBO may also be an underlying cause of the symptoms. Our meta-analysis did not find evidence that different detection methods for SIBO might affect HP infection and the prevalence of SIBO in adults. However, it is important to interpret these results cautiously since only one or two datasets were included for the subgroups with LBT and QDC. Moreover, efforts are still needed to determine the optimal methods for detecting SIBO [33]. Notably, it may be interesting to determine the relationship between high-resolution methods detected by HP infection [34] and SIBO validated with the multi-omics approach, especially metabolomics [35]. Finally, a more clinically relevant question is determining the optimal treatment for patients with HP infection and SIBO. A recent study showed that eradicating HP infection with the quadruple regimen containing amoxicillin and metronidazole was associated with an improved remission of SIBO [30]. Studies are needed to determine if treatment for SIBO is necessary after HP eradication for patients with digestive symptoms.

This study has limitations. First, observational studies provided the basis for the meta-analysis. Many factors may confound the association between HP infection and SIBO, including factors affecting intestinal microbes, such as dietary factors. Second, since this was a meta-analysis of observational studies, a causal relationship between HP infection and SIBO could not be determined. Future studies should also analyze the correlation between HP infection and SIBO and their relationship to digestive symptoms. However, our meta-analysis has a limited sample size, and its results should be validated in future studies.

## Conclusions

Overall, the meta-analysis results suggest that HP infection is associated with a higher prevalence of SIBO in adults, especially younger individuals. The detection of SIBO should be considered for patients with digestive symptoms and HP infection. Furthermore, there is a need to determine whether eradicating HP can reduce SIBO in patients with this condition.

## Methods

Throughout the process of planning, conducting, and reporting the study, the Preferred Reporting Items for Systematic Reviews and Meta-Analyses statement [36, 37] and Cochrane Handbook [38] were followed.

### Search of databases

We searched electronic databases, including PubMed, Embase, Cochrane Library, and Web of Science, starting inception and ending March 5, 2023, for studies published by that date. The search was performed with the terms including (1) “HP” OR “H. pylori” OR “Helicobacter pylori” and (2) small intestinal bacterial overgrowth” OR “small intestine bacterial overgrowth” OR “small bowel bacterial overgrowth” OR “SIBO” OR “SBBO.“ There was no limitation on the language of the publication in the search for human studies. As part of our manual screening process, references from relevant original and review articles were screened for possible relevant studies.

### Inclusion and exclusion criteria of studies

Inclusion criteria were developed per the PICOS recommendations and according to the aim of the meta-analysis.

#### P (patients)

Adult patients who took tests for HP infection and SIBO without recent antibiotic exposure.

#### I (exposure)

Patients with HP infection. Methods used for validating HP infection were consistent with those used in the original study, which mainly included the urea breath test (UBT), stool antigen test (SAgT), and the rapid urease test (RUT) during upper gastrointestinal endoscopy.

#### C (control)

Patients without HP infection.

#### O (outcomes)

SIBO prevalence compared between patients with and without HP infection. Studies included in this review applied consistent methods and criteria for detecting SIBO, which mainly included the lactulose breath test (LBT), the glucose breath test (GBT), and the quantitative duodenal aspirate culture (QDC).

#### S (study design)

Observational studies, which included case-control studies, cross-sectional studies, and cohort studies.

Reviews, editorials, studies including children, studies that did not evaluate HP infection or SIBO, or studies that reported a history of HP infection rather than current HP infection were excluded. In cases of overlap in patient populations, the study with the largest sample size was included in the meta-analysis.

### Data extraction and quality evaluation

Two authors carried out literature searches, data collection, and study quality assessments independently. In case of discrepancies, a third author was contacted for a discussion to reach a consensus. Among the studies included in the analysis, we collected information regarding study information, demographic factors, diagnosis, and methods for diagnosing HP infection and detecting SIBO. In terms of quality, the study was scored using the Newcastle–Ottawa Scale [39] based on the criteria for participant selection, the comparability of the groups, and the validity of the outcomes. Nine stars were on the scale, with a larger number representing a better study.

### Statistics

A total number of patients with SIBO in participants with and without HP infection was derived for all studies included in this analysis. Statistical analyses of the association between HP infection and SIBO in these patients were performed using odds ratios (ORs) and their corresponding 95% confidence intervals (CIs). In order to estimate between-study heterogeneity, the Cochrane Q test and the I^2^ statistic [40] were used. An I^2^ > 50% indicates that there is significant heterogeneity between studies. A random-effects model was applied if there was significant heterogeneity; otherwise, a fixed-effects model was applied [38]. To evaluate how individual studies affected meta-analysis results, the sensitivity analysis excluded one dataset at a time [41]. In order to determine the influence of study characteristics on the outcome, subgroup analyses were performed according to the study country, comorbidities, possible exposure to proton pump inhibitors (PPIs), mean ages of the patients, and methods for detecting HP infection and SIBO. For subgroup analysis, according to a continuous variable, the median of the variable was selected as cutoff for defining subgroups. A funnel plot is used to estimate publication bias based on visual judgments of symmetry, along with Egger’s regression asymmetry test [42]. The statistical analyses were carried out with RevMan (Version 5.1; Cochrane Collaboration, Oxford, UK) and Stata software (version 12.0; Stata Corporation, College Station, TX).

## Data Availability

All data generated or analyzed during this study are included in this published article.

## Abbreviations

SIBO: small intestinal bacterial overgrowth
GI: gastrointestinal
IBS: irritable bowel syndrome
HP: *Helicobacter pylori*
UBT: urea breath test
SAgT: stool antigen test
RUT: rapid urease test
LBT: lactulose breath test
GBT: glucose breath test
QDC: quantitative duodenal aspirate culture
ORs: odds ratios
CIs: confidence intervals
PPIs: proton pump inhibitors
